# Effects of Dietary Biological or Chemical-Synthesized Nano-Selenium Supplementation on Growing Rabbits Exposed to Thermal Stress

**DOI:** 10.3390/ani10030430

**Published:** 2020-03-04

**Authors:** Asmaa M. Sheiha, Sameh A. Abdelnour, Mohamed E. Abd El-Hack, Asmaa F. Khafaga, Khaled A. Metwally, Jamaan S. Ajarem, Saleh N. Maodaa, Ahmed A. Allam, Mohamed T. El-Saadony

**Affiliations:** 1 Department of Animal Production, Faculty of Agriculture, Zagazig University, Zagazig 44511, Egypt; asmaasheiha76@gmail.com (A.M.S.); samehtimor86@gmail.com (S.A.A.); 2 Department of Poultry, Faculty of Agriculture, Zagazig University, Zagazig 44511, Egypt; 3 Department of Pathology, Faculty of Veterinary Medicine, Alexandria University, Edfina 22758, Egypt; 4 Department of Soil and Water Sciences, Faculty of Technology and Development, Zagazig University, Zagazig 44511, Egypt; khametwally@zu.edu.eg; 5 Department of Zoology, College of Science, King Saud University, P.O. Box 2455, Riyadh 11451, Saudi Arabia; jajarem@ksu.edu.sa (J.S.A.); maodaa_28@yahoo.com (S.N.M.); 6 Department of Zoology, Faculty of Science, Beni-suef University, Beni-suef 65211 Egypt; allam1081981@yahoo.com; 7 Department of Agricultural Microbiology, Faculty of Agriculture, Zagazig University, Zagazig 44511, Egypt; m_tlatelsadony@yahoo.com

**Keywords:** nano-selenium, biological synthesis, heat stress, growth, antioxidants

## Abstract

**Simple Summary:**

This study was designed to compare the potential effects of nano-selenium synthesized by biological or chemical methods on growth, carcass, oxidative, and inflammatory parameters of growing rabbits reared under thermal stress. Graded levels were used from both selenium sources (0, 25, and 50 mg/kg diets). The biologically synthesized nano-selenium showed superior improvement effects for the studied parameters compared to the chemically synthesized nano-selenium in terms of alleviating heat stress effects and improving growth, carcass, oxidative, and inflammatory parameters of growing rabbits.

**Abstract:**

The adverse influences of elevated ambient temperatures during the summer season on the rabbit industry have received increased global attention. Therefore, this study intended to compare the potential effects of nano-selenium (nano-Se) synthesized by biological (BIO) and chemical (CH) methods on growth performance, carcass variables, serum metabolites, and inflammatory cytokines responses of growing rabbits in the summer season. Two hundred and fifty weaned rabbits (males, 35 days of age) were randomly divided into five treatment groups of 50 rabbits each (each group had five replicates with ten male rabbits). Treatment groups were fed a control diet and four controlled diets supplemented with nano-Se synthesized by biological method (BIO25 and BIO50, with a 25 and 50 mg of nano-Se/kg diet, respectively) and chemical method (CH25 and CH50, with a 25 and 50 mg of nano-Se/kg diet, respectively) for eight weeks. During 11 to 13 weeks of age, a gradual enhancement in live body weight (LBW), feed intake (FI) and feed conversion ratio (FCR) was noticed with BIO25 and BIO50 treatments compared to those in the other groups. The carcass percentage was significantly higher (*p* < 0.01) for animals fed with BIO25 than the other groups. The other organ functions were significantly higher (*p* < 0.01) in heat-stressed groups compared to that of nano-Se groups. Increasing the level of only BIO from a 25 to a 50 mg/kg diet gave more improvement in the studied parameters. Additionally, the concentrations of serum urea, triglycerides (TG), and glutamyl transferase (GGT) were lower (*p* < 0.01) in both treated and untreated groups. Likewise, the supplementation with nano-Se (BIO25, BIO50, or CH25) significantly improved the antioxidant indices and inflammatory cytokines responses as indicated from serum metabolites. Based on the study results, nano-Se especially synthesized by the biological method at diet levels of 25 or 50 mg/kg improved the growth performance, kidney and liver functions, carcass traits, antioxidants indices, and inflammatory cytokines of growing rabbits during thermal stress.

## 1. Introduction

Currently, global warming represents a main menace not only for human health but also for different species of livestock. In Egypt, through the hot months, the elevated ambient temperature is exacerbated by the high relative humidity which is normally over 85% during the day and can reach 100% during the night [1,2,3,4]. Recently, the rabbit industry, as a source of animal proteins, has shown an increasing role in the national economies of in Mediterranean regions [5]. However, rabbits are very sensitive to heat stress due to an absence of sweat glands in their skin, except the ear area [4]. Increased oxidative stress induced by thermal stress has been accompanied with significant reduction in the antioxidant defense in the body, inducing inflammatory responses and decreasing feed intake and growth rate, leading to economic losses in the rabbit industry [1,4,6,7].

Several approaches have been developed to avoid the deleterious impacts of thermal stress in animals including the use of phytogenic feed additives, minerals, and vitamins [1,2,3,7]. Hence, numerous kinds of microelements are used effectively to decrease the injurious effects of heat stress in several animal species and even to avoid their adverse impacts [1,2]. Selenium (Se) is a microelement indispensable in animal health and feeding. It exhibits multiple actions linked to animal production, fertility, and disease prevention [8,9]. Selenium aids the antioxidant enzymes in the body and in turn helps control levels of lipid peroxides and hydrogen peroxide that are formed through ordinary metabolic activity [10]. Likewise, dietary Se is vital for immune system activity, particularly during adverse environmental stressors [10,11,12]. The bioavailability of selenium is related to its physical form [11]. With the recent progress of the nanotechnology field, nano-selenium (nano-Se) has acquired extensive attention because the nanometer particles display innovative features such as a high surface activity, high catalytic efficiency, large surface area, strong adsorbing ability, and low toxicity [13,14,15]. Recently, nano-Se has been widely used in poultry nutrition. However, there is a huge concern about the application of the chemical form of nano-Se [16]. The chemical technique can cause hazardous effects for the environment and animals [13,15]. More recently, biosynthesis of nanoparticles is a new tactic for synthesizing nanoparticles that uses plants and microorganisms which have biomedical uses [16,17,18]. This approach is environmentally friendly, safe, easy to obtain, biocompatible, and cost effective [17].

Hence, we hypothesized that maintainable supports for dietary manipulation constructed on nano-Se supplementations are mainly desirable, especially when reflecting the additional benefits of nano-Se such as cost effectiveness, actual efficiency, and availability. Recently, many reports about different biomedical applications of selenium nanoparticles have been published [18,19]. No investigations have been stated to compare the probable effects of nano-Se synthesized by green or chemical methods in growing rabbits. Therefore, this study was conducted to investigate the potential effects of chemical or biological synthesized nano-Se on growth efficiency, carcass traits, serum biochemicals, antioxidant status, inflammatory responses, and cecal microbial populations in growing rabbits exposed to thermal stress.

## 2. Materials and Methods

This study was carried out at the Rabbit Research Unit, Faculty of Agriculture, Zagazig University, Egypt. The experimental procedures regarding the handling and care of rabbits were approved by the Institutional Ethics Committee of the Zagazig University, Zagazig, Egypt. All procedures by this study were in accordance with international ethical standards. The research involved no human participants.

### 2.1. The Source of SeleniumU in this Study

Firstly: biological selenium (BIO). This was synthesized from lactic acid bacteria that were isolated from human breast milk. Optimized conditions were created for the synthesis and characterization of the nano-Se. Secondly: chemical selenium (CH). The selenium nanoparticles were synthesized by using the wet chemical approach described by Qian et al. [20].

### 2.2. Antibacterial Activity of Nano-Se

The antibacterial activity of nano-Se was assessed by agar well diffusion. Three strains of Gram-positive bacteria were used, i.e., *Bacillus cereus* (ATCC 21366), *Listeria monocytogenes* (ATCC 7644), and *Staphylococcus aureus* (ATCC 29213), and three Gram-negative strains, i.e., *Escherichia coli* (ATCC 25922), *Salmonella Typhimurium* (ATCC 39183), *Klebsiella pneumoniae* (ATCC 13883), and *Pseudomonas aeruginosa* (ATCC 27853) bacteria. The strains were grown in Mueller–Hinton agar slants overnight at 35 °C. The pure cultures of all the established pathogens were inoculated to fresh nutrient broth (NB) in discrete tubes. Different levels of nano-Se (25, 50, 75, and 100 µL of either biological nano-Se (BIO) or chemical nano-Se (CH) solution) and oxytetracycline and colistin as controls were transferred to each plate well. After incubation at 37 °C for 24 h, the appeared levels of zone of inhibition were measured and interpreted using the zone diameter interpretive standards.

### 2.3. Animals and Experimental Groups

A total of 250 weaned male rabbits with an average weight of 666.8 g ± standard deviation (35.8 g) and 35 days of age were used in the experiment. Animals were randomly divided into five treatments (50 animals in each treatment, each with five replicates (ten rabbits each)). The experiment lasted for eight weeks during summer conditions in Zagazig (30°34′ N, 31°30′ E), Egypt. Growing rabbits were fed the basal diet with nano-selenium (nano-Se) synthesized by biological (BIO) or chemical method (CH). Diet formulation was produced to meet growing requirements according to the National Research Council (NRC). The treatments were as follows: (1) control basal diet (CO); (2 and 3) basal diet + (25 or 50 mg) of biological nano-selenium/kg (BIO25 and BIO50); (4 and 5) basal diet + (25 or 50 mg) of chemical nano-selenium/kg (CH25 or CH50). Throughout the experimental period, animals were kept independently in galvanized wire cages (length 35, width 35, and height 60 cm). All animals were housed under similar environmental, management, and hygienic conditions. Throughout the investigational period, each cage enclosed a pot and feeder to offer free access to fresh water and feed, respectively. Every morning, feces and urine on the rabbit tray floor were removed. Rabbits were exposed to normal light throughout the experimental period. A chemical analysis (see the table in the Supplementary Materials) of the diet showed 17.5% crude protein, 7.95 MJ metabolizable energy, 0.88% calcium, and 0.20% available phosphorous [21].

### 2.4. Temperature Humidity Index (THI) Calculation

For estimating the THI values, which indicate the degree of heat stress in rabbits, ambient temperatures and relative humidity were estimated throughout the study period using an automatic thermo hygrometer (OF 14:140, H 10–99%; TFA Dostmann GmbH and Co. KG, Wertheim, Germany) in the afternoon (14:30 p.m.). The THI was considered conferring to LPHSI [22] as the subsequent calculation: THI = db°F − [(0.55–0.55RH) (db°F − 58)], where RH is the relative humidity as a percentage, and db°F is dry bulb temperature in Fahrenheit degrees. The values of THI attained were then characterized as follows: <82 (absence of heat stress), 82 ≤ 84 (moderate heat stress), 84 ≤ 86 (severe heat stress, and 86 and more (very severe heat stress). These values followed calculations recorded by Marai et al. [23] in small animals (rabbits and poultry).

### 2.5. Growth Performance

Growth performance traits were taken weekly during the experimental period. These were live body weight (LBW), feed intake (FI), body weight gain (BWG) and feed conversion ratio (FCR).

### 2.6. Carcass Measurements

At the end of the experimental period (13 weeks of age), nine animals from each treatment were randomly chosen and fasted for 12 h before the time of slaughtering. The animals were individually weighed and slaughtered conferring to Islamic procedures. After bleeding, animals were weighed and skinned. The carcasses were weighed before and after removal of viscera [24]. The lungs, heart, liver, kidneys, and spleen were weighed and expressed as relative weight from the live weight of the rabbits.

### 2.7. Serum Biochemical, Inflammatory and Antioxidants Indices

The blood samples were taken from the slaughtered animals in non-heparinized sterile tubes. The samples were allowed to coagulate at room temperature for 30 min and then centrifuged for 15 min at 3500 rpm for serum collection. Serum samples were preserved at −20 °C for further analysis. Serum contents of creatinine, urea, total triglycerides, total bilirubin, and direct and indirect bilirubin were assessed conferring to the manufacturer’s instructions by the spectrophotometric technique using commercial diagnostic kits (Biodiagnostic Co., Giza, Egypt). Serum lysozyme activity of the experimental rabbits was estimated via the turbidometric method [25]. Lipid peroxidation was evaluated through the determination of malondialdehyde (MDA) [26]. Serum contents of superoxide dismutase (SOD), reduced glutathione (GSH) and catalase (CAT) were assessed by spectrophotometric procedures (Hitachi spectrophotometer, Tokyo, Japan) using commercially available kits (Biodiagnostic Co., Giza, Egypt).

### 2.8. Microbiological Analyses

Ten grams of rabbit cecum from each replicate (5 samples per each treatment) were separately transferred to a 250 mL Erlenmeyer flask containing 90 mL of sterile peptone saline solution (0.1% peptone and 0.85% NaCl) and well mixed, then serial dilutions up to 10^7^ were prepared. The total bacterial count, *Enterococcus* and total coliforms were enumerated according to Harrigen and Mccance-Margart [27]. The total coliforms were counted according to Kreger-van [28] using MacConkey agar medium. To identify *E. coli* biochemical assays of indole, methyl red, Voges-Proskauer and citrate reactions were done. *Salmonella* and *Shigella* spp. were counted using S.S. agar (Oxide CM 99). Black and pink colonies on S.S. agar are typical colonies of *Salmonella* spp. and *Shigella* spp. Molds were enumerated by using potato dextrose agar (PDA) medium at 28 °C for 72 h [28].

### 2.9. Statistical analysis

Traits were statistically explored using SPSS^®^ software program version 21 (SPSS, Chicago, IL, USA). The growth performance, serum metabolites, antioxidants, inflammatory response, and carcasses traits were assessed with a one-way analysis of variance (with the diet as the fixed factor) using the post-hoc Newman–Keuls test. Differences were statistically considered significant at *p* < 0.05. The statistical model used was:

Y_ijk_ = μ + T_i_ + e_ijk_ (1)

where Y is either the trait; μ is the mean of the trait, T_i_ is the effect of supplementation level and e_ijk_ is the error respectively.

## 3. Results

### 3.1. Antibacterial Activity of Nano-Se

As presented in Table 1, the various sources of nano-Se showed strong antibacterial activity against six pathogenic bacteria: *B. cereus*, *L. monocytogenes*, *S. aureus*, *E. coli*, *Salmonella Typhimurium*, and *K. pneumoniae*. In general, the obtained results showed that nano-Se obtained by biological method is more effective in the inhibition of pathogenic bacteria than nano-Se obtained by the chemical technique (Table 1). The study indicated that the effect of nano-Se was found to be more definite with *Staphylococcus aureus* and *Salmonella Typhimurium* when compared to the other pathogens (Table 1). Moreover, the inclusion of nano-Se (BIO) with two types of antibiotics enhanced the efficiency of antibiotics against pathogens. In the case of all the bacteria, the incorporation of oxytetracycline or colistin with nano-Se (BIO) displayed an increase in the zones of inhibition compared to oxytetracycline or colistin alone. The minimum inhibitory (MIC) and minimum bacterial concentration (MBC) against the explored pathogen bacteria were up 160 µg/mL for *Staphylococcus aureus*, *E. coli*, and *K. pneumonia,* while the MIC and MBC were up 80 µg/mL for *B. cereus, L. monocytogenes*, and *S. Typhimurium.* This feature of nano-Se (BIO) indicates its bacteriostatic and bactericidal activities (Table 2).

### 3.2. Temperature Humidity Index (THI) Values

**Figure 1:**
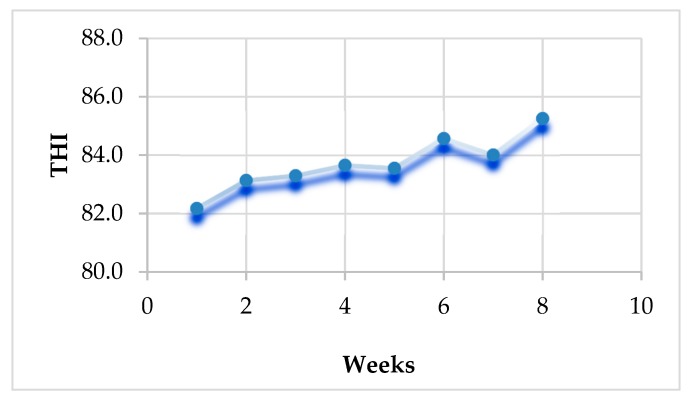
Calculated temperature humidity index (THI) throughout the experimental period.

Results presented in Figure 1 show that during the first five weeks of the experiment, the calculated THI values ranged between 82.17 and 83.65, elucidating moderate heat stress. After that, during the last three weeks of the study, the estimated THI values ranged from 84.01 to 85.25 representing a severe degree of heat stress on the animals. Likewise, the overall mean of THI values throughout the experiments was 84.07, reflecting a state of initiation of severe heat stress.

### 3.3. Growth Performance

Both levels of BIO (25 or 50 mg/kg) significantly improved the LBW of growing rabbits exposed to heat stress compared with CH and control groups at the first week and last three weeks of the experiment. At the end of the experiment, LBW was significantly increased (*p <* 0.05) by about 14.23% and 8.50%, in BIO50 compared with CH50 and the control group, respectively. Additionally, no significant changes were detected among the treated groups (except BIO50) and the control in the final live body weight. However, LBW was improved in BIO25, BIO50, and CH25 treatments compared to the control group.

Regarding daily body weight gain (DBWG; g/day), the highest values of DBWG with significant differences at the end of study were observed (*p <* 0.01) in both BIO25 and BIO50 treatments compared with the other treatments (Table 3). However, no significant variances were demonstrated among treated groups (except BIO50) and the control after 5 weeks of the experiment. After 6 weeks of the study, it was interesting to observe that CH25 treatment showed an increase (15.50%) of BWG followed by BIO50 (8.91%) in relation to the non-treated group.

At five weeks of age, the BIO25 treatment decreased (*p <* 0.05) feed intake (FI) compared to the other treatments. However, no significant differences were observed among the treated groups (except CH50) and the control (Table 3). The biosynthesis of nano-Se either BIO25 or BIO50 under heat stress resulted in a significant improvement in FI by 23.75% and 16.25%, respectively, compared with the control. On the other hand, the CH25 and CH50 induced significant reduction in feed intake at the end of the experiment by about 7.50% and 15.00%, respectively, compared with the control.

With regards to the feed conversion ratio (FCR), green biosynthesis of nano-Se (BIO25 and BIO50) and CH25 significantly improved *(p <* 0.01) the FCR in heat-stressed rabbits compared with non-treated and CH50 groups.

### 3.4. Carcass Traits

The BIO25 group showed significant enhancement in carcass percentage (*p <* 0.001) compared with the other groups However, no significant differences were found among the BIO50, CH25, or CH50 groups (Table 4). Both the control and the BIO25 group resulted in significant increase (*p <* 0.001) in the liver percentage in comparison to the other treated groups during the exposure to thermal stress. Values of lung percentages were higher (*p <* 0.05) in the CH (CH25 or CH50) groups and the control groups compared to the biological nano-Se groups. On the other hand, heart percentages recorded the highest values in the control and BIO (25 or 50) treatments compared with CH treatments. The percentages of kidneys and spleen were not influenced by the addition of different forms of nano-Se, however, they were greater in nano-se synthesized by the chemical method than the biological method.

### 3.5. Blood Serum Metabolites

The BIO50 treatment significantly decreased (*p <* 0.01) serum urea content compared to the other treated groups and the control. The highest values were recorded with the control, while the BIO25, CH25, and CH50 treatments gave intermediate values. The treatments of BIO50 and CH25 produced the lowest values of GGT (*p <* 0.01) and TG (*p =* 0.05) in the serum of heat-stressed rabbits. Moreover, the highest values of GGT and TG were reported in the CH50 and control groups, respectively. All of the experimental additives did not exhibit any significant effect on the creatinine, total bilirubin, or direct and indirect bilirubin contents in the serum of heat-stressed rabbits (Table 5).

### 3.6. Inflammatory Responses

The administration of nano-Se during the heat stress period (BIO25, BIO50, and CH25) for eight weeks decreased the production of interleukin 4 (IL-4) (*p* = 0.01) compared to the control and CH50 groups (Table 5). Similar to IL-4, the interferon-gamma (IFNγ) was reduced (*p* = 0.001) by the nano-Se (except CH50). The control group exhibited intermediate values. On the other hand, a significant increase in the lysozyme activity (LZM) was observed in the BIO50 and CH25 groups compared to the other groups (Table 5).

### 3.7. Antioxidant Indicators

The highest values of antioxidative indicators were observed in the control group. The other treated heat-stressed rabbit by nano-Se (except CH50) had significant reduction in values of nitric oxide (NO) and MDA production compared the control group. The SOD levels were significantly decreased by nano-Se supplementation. The BIO25 and BIO50 groups gave lower values of SOD than the other groups (Table 5). With regards to GSH, the CH25 treatment recorded the highest values (*p <* 0.001) of GSH followed by the BIO25 and BIO50 treatments compared to the control and CH50 groups. Similar improvement was noticed for the CAT levels in growing heat stressed-rabbits after treatments with nano-Se (CH25, BIO25, and BIO50).

### 3.8. Impact of Nano-Se (BIO or CH) on Cecal Microbiota

**Figure 2:**
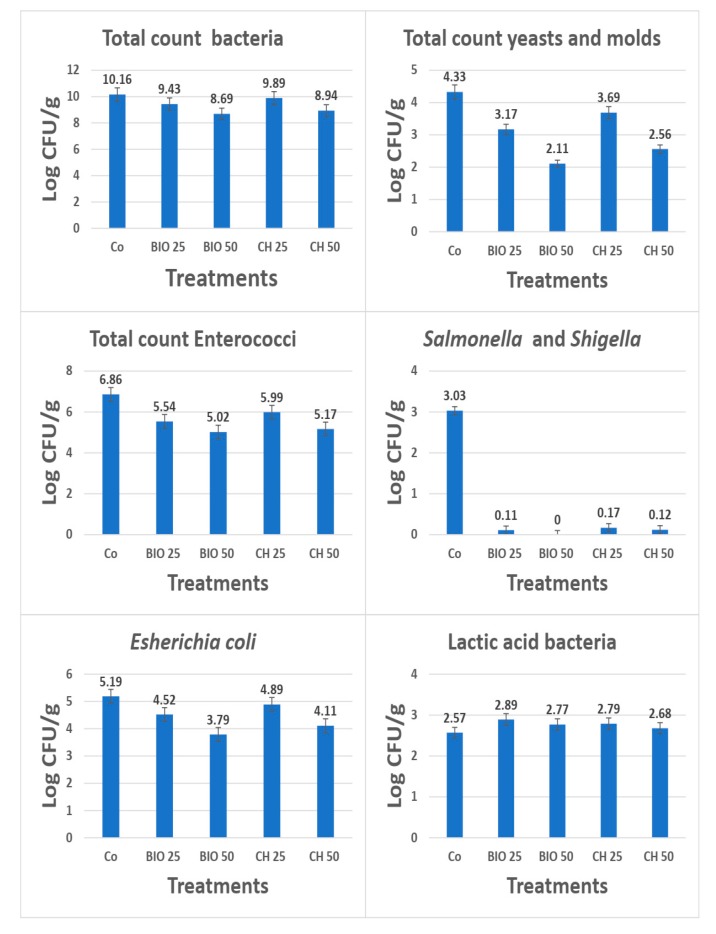
Changes in bacterial count (log cfu/g) determined in cecum of rabbit.

The microbial load in the rabbit cecum decreased by increasing the feed additive concentration. This trend was the same for the six tested microbial groups (bacteria, fungi, *Enterococci*, *Coliform*, *Salmonella* and *Shigella* and *Staphylococi*) (Figure 2). The incorporation of 0.25 and 0.50 mg of biological nano-Se eliminated both *Salmonella* and *Shigella* in rabbits exposed to thermal stress. Additionally, the 0.25 mg of biological nano-Se increased the counts of lactic acid bacteria in the cecum of rabbits. The biologically synthesized nano-Se was more effective than the chemically synthesized. From the results obtained, decreases in the total count of bacteria as well as pathogenic bacteria were observed (Figure 2).

## 4. Discussion

Global warming has been identified as a factor that potentially contributes to the reduction of productivity and economic profit in the rabbit industry. The use of dietary manipulations is an accepted approach that overcomes the adverse influences of thermal stress in rabbits [7,29]. These include dietary minerals, vitamins, essential oils, amino acids, and phytogenic feed additives [2].

Presently, elemental nano-Se has gained interest owing to its low toxicity, high adsorbing ability, high bioavailability, and high catalytic efficiency in comparison with selenite in chickens [30], sheep [31], and goats [32]. There are several methods for synthesis of nano-Se—chemical methods of nano-Se have been widely used in animals, however, there is a huge concern about the application of chemical forms of nano-Se.

Numerous microorganism strains can decrease the toxic selenite oxyanion to the less toxic elemental selenium through the biotransformation of either intracellular or extracellular nano-Se, with a typical spherical shape and a diameter of 50–400 nm [18]. Additionally, recent studies with nano-Se (BIO) have established that the particles have anti-biofilm activity against medical isolates of bacterial pathogens [19]. These results may be attributed to the smaller diameters of biologically manufactured nano-Se compared to the chemically manufactured nano-Se. Generally, the biological nano-Se at levels of 50–100 µg/mL was more effective in the destruction of existing biofilm constructions than the inhibition of biofilm formation. In this context, nano-Se synthesized by biological techniques possesses antibacterial, antiviral, and antioxidant properties, signifying they could be suitable as therapeutic candidates to combat infectious diseases.

In the present study, according to the estimated values of THI (84.07 on average), growing rabbits were suffering from severe heat stress during the experimental period. However, distinct betterment in growth performance, carcass, antioxidants, and inflammatory responses were detected as a consequence of nano-Se (BIO25, BIO50, CH25, and CH50) dietary supplementation.

The present research shows that feeding different sources of nano-Se was useful in improving LBW, BWG, FI, and FCR in heat-stressed rabbits compared to the control. Several studies confirmed the ability of a nano-Se diet (0.2–0.3 mg/kg) to remarkably improve the BWG [33] and FCR [33,34] in poultry under heat stress conditions. Similar improvement in growth performance was obtained by Shi et al. [31], who concluded that diets supplemented with nano-Se resulted in enhanced final body weight, BWG, and FCR in guangxi yellow chicken. Boostani et al. [8], reported significant improvement in the BWG of broilers fed diets supplemented with 0.2 mg/kg of organic Se as compared with inorganic Se and the control groups.

Abdel-Wareth et al. [35] reported significant enhancement in final BW, FCR, and daily BW gain in heat-stressed rabbits supplemented with nano-Se compared to the non-supplemented group. However, feed consumption was not significantly affected by the treatments. In the current study, heat-stressed rabbits supplemented with a biological nano-Se diet (25 or 50 mg/kg) had higher BWG, FC, and FCR compared with the non-supplemented diets. The positive effects of biological nano-Se might be due to its antioxidant activities that enhance the digestion and absorption of nutrients [17]. In accordance with our results, Zhang et al. [36] reported an enhancement in the growth performance of rabbits fed diet supplemented with Se 0.70 mg/kg. In parallel, Zeweil et al. [37] reported that adding different sources of Se to rabbit diets significantly reduced FI and enhanced FCR from 11.5% to 19.4% owing to different treatments compared to the control.

In contrast with our results, previous studies have indicated that dietary Se supplementation did not have any influence on the overall broiler performance [34,38]. Additionally, El-Deep et al. [34] indicated that the addition of nano-Se (0.3 mg/kg) into laying hens’ diet did not affect the FI during high environmental temperatures. This might confirm the ability of nano-Se, especially that which is synthesized by biosynthesis method at low levels (25 or 50 mg/kg), to improve the growth performance of growing rabbits in a hot environment through increasing the LBW, BWG, FI, and FCR. The results of the present experiment revealed that the carcass percentage was significantly improved by supplementing the diets with biological nano-Se at a level of 25 mg/kg followed by both BIO50 and CH50 treatments compared with the heat-stressed group.

The administration of nano-Se (BIO50 or CH25) reduced blood urea level compared to the control, while the values of GGT and TG for rabbits fed nano-Se (BIO25, BIO50, or CH25) were lower than those of the control group at the end of the experiment. Concerning liver and kidney functions, creatinine and total, direct and indirect bilirubin did not exhibit any significant changes among the groups. Similar to our results, Abdel-Wareth et al. [35] showed that rabbits fed with nano-Se had a significantly lower urea level compared to the control group. Additionally, a significant reduction was reported in the TG level as a response to nano-Se supplementation in broiler chicks [38]. These results concluded that rabbits fed nano-Se had improved liver and kidney functions, thus alleviating the adverse influences of a hot environment. Nano-Se can improve the lipid profile which could be closely connected to the protective roles against heat stress. Moreover, the potential role of selenium-coated nanostructures has also been revealed to be effective in the treatment of fatty liver disease, by enhancing the oral bioavailability and strengthening the hypoglycemic action of phytogenesis [16].

Our results showed significant decrease in the inflammatory cytokines (IFNγ and IL-4) of rabbit serum as affected by nano-Se supplementation. Heat stress could increase the generation of cellular reactive oxygen species (ROS), which leads to protein degradation and further decline in protein synthesis [1,39]. Excessive production of ROS results in augmented lipid peroxidation, cellular damage, oxidative stress, and inflammatory responses [1,40]. Pro-inflammatory cytokines (e.g., IL or TNF-γ) are supposed to be responsible for the detrimental effects of a hot environment. During the exposure to high temperatures, the secretions of cytokines was observed to be increased in animals, suggesting heat load on the animals which can further cause fever, reduce the appetite, and increase energy expenditure and fat mobilization [1,2,4]. The present study shows a reduction in inflammatory cytokines function in rabbits fed nano-Se compared to the control, which reflects the efficacy of nano-Se to enhance animal heath in a hot environment.

Enhancing animal resistance during adverse environmental stress is an important approach to reduce or avoid several infectious disorders via persuaded immune enhancers and stimulants [2,41]. Nano-Se as feed additives is used in the diets for this purpose. It has been indicated that lysozyme activity is a principal innate immune defense index and hyperthermia might alter the lysozyme activity. In the current study, dietary supplementation of nano-Se (BIO50 or CH25) significantly increased serum lysozyme activity, indicating nanoparticles can improve the innate immune defense of heat stress exposed rabbits.

The boost in antioxidant enzyme activities is considered as a protective response against oxidative stress which is generated by heat injury. In our study, nano-Se supplementation in rabbit diets enhanced the antioxidant activities by significantly increasing the activities of GSH and CAT compared to those in the heat stress group, and significantly reduced both NO and MDA levels in the nano-Se treated groups (BIO25, BIO50, or CH25) compared to other groups. It has been reported that dietary inclusion of nano-Se (0.2–0.3 mg/kg) in poultry significantly improved the activity of reduced glutathione (GSH-P) in the serum [34]. Consequently, the immunological activities and protective role of Se, which have been enhanced by the nano-Se addition definitely could alleviate the negative influences on heat-stressed laying hens. Moreover, Zhou et al. [42] showed that Se contributes to several immune functions at all levels, such as minimizing levels and duration of inflammatory infections, reduction of glucocorticoids (indicator for immunity disorder), arrangement of the function of T lymphocytes cells and T killer cells, and activation of IL-2.

In broilers, Boostani et al. [8] reported that Se (0.3 mg/kg nano-Se) supplementation significantly augmented the levels of glutathione (GSH) and glutathione peroxidase (GSH-Px) activity in comparison with the control group. A detailed examination showed the vital role of Se in antioxidant defense. Se is a vital trace element for combating oxidative stress, and hence the redox state of the cell, because of its integration as selenocysteine into GSH-Px [37] and thioredoxin reductase [1]. In the present work, diet supplementation with nano-Se, (CH25, BIO25, and BIO50) increased the GSH-Px activity in heat-stressed rabbits. This suggests the potential role of nano-Se to overcome the negative impacts of heat stress compared with the control and the high level nano-Se synthesized by chemical method (CH50) group.

MDA is a sensitive biomarker of lipid peroxidation in the cells. Hence, the higher MDA concentrations observed in heat-stressed rabbits strongly show that the period of heat was indeed stressful to these animals and had undesirable impacts on immune responses [1,43]. Co-administration to heat-stressed rabbits of nano-Se synthesized by biological method had the effect of reducing the NO and MDA production compared with the CH50 and the control groups. Similarly, nano-Se addition into birds’ diets exhibited lowered MDA levels compared to control and stressed birds [8]. The efficiency of nano-Se in the elimination of pathogenic microbes in the digestive tract of animals leads to the creation of a suitable environment for microbes that make up the natural flora found in the stomach and intestines, and enables them to perform their functions more efficiently (Figure 2). The high effectiveness of green synthesis of nano-Se in eliminating pathogens, which provides an appropriate environment for lactic acid bacteria as well as probiotic bacteria, improves the metabolism of animals. Probiotics can provide benefits to the host’s intestine through a variety of mechanisms that include competitive exclusion of pathogens, production of antimicrobial compounds, intestinal detoxification, modification of the host’s immune responses, and maintenance of the intestinal barrier [44]. Furthermore, suppression of pathogenic microbes might positively support growth and metabolism of beneficial microorganisms and therefore reflects as improved rabbit performance, as observed in this study.

## 5. Conclusions

In view of the above findings, nano-Se is a vital element that helps growing rabbits to cope with the negative impacts of heat stress through many physiological processes. Dietary nano-Se synthesized by biological method at 25 or 50 mg /kg in rabbit diets during heat stress resulted in enhanced growth performance, liver and kidney functions, and antioxidant status and regulated the inflammatory cytokines responses. It is a good and beneficial approach to alleviating the deleterious impacts of thermal stress on growing rabbits. The results showed that the high levels of nano-Se synthesized by chemical method had negative impacts on the antioxidants parameters of growing rabbits.

## Figures and Tables

**Table 1:** In vitro anti-activity of nano-Se (CH or BIO) against several types of bacteria (inhibition zone, mm).

Items	Average Diameter of the Inhibition Zone (mm)											
	**25 µL**		**50 µL**		**75 µL**		**100 µL**		**Synergism with Antibiotic**			
	**Nano-Se CH**	**Nano-Se BIO**	**Nano-Se CH**	**Nano-Se BIO**	**Nano-Se CH**	**Nano-Se BIO**	**Nano-Se CH**	**Nano-Se BIO**	A	B	C	**D**
*B. cereus*	23	25	27	28	29	31	30	34	27	30	31	35
*L. monocytogenes*	24	26	26	29	27	32	29	33	28	31	32	36
*S. aureus*	22	26	25	27	26	30	28	32	26	28	30	35
*E. coli*	19	21	20	22	21	24	23	25	24	25	26	29
*S. Typhimurium*	21	25	24	27	26	29	28	30	28	31	27	31
*K. pneumoniae*	20	21	22	24	23	26	26	29	25	27	25	30

Nano-Se CH: chemical synthesis of selenium nanoparticles, nano-Se BIO: biological synthesis of selenium nanoparticles, (A): oxytetracycline only (B): oxytetracycline + nano-Se BIO.

**Table 2:** Minimum inhibitory concentration (MIC) and minimum bacterial concentration (MBC) of biological nano-Se. (C): colistin only (D): colistin + nano-Se BIO.

Test Pathogenic Bacteria	20 µg/mL	40 µg/mL	80 µg/mL	160 µg/mL	320 µg/mL	640 µg/mL
*B. cereus*	+	+	ـــ	ـــ	ـــ	ـــ
*L. monocytogenes*	+	+	ـــ	ـــ	ـــ	ـــ
*S. aureus*	+	+	+	ـــ	ـــ	ـــ
*E. coli*	+	+	+	ـــ	ـــ	ـــ
*S. Typhimurium*	+	+	ـــ	ـــ	ـــ	ـــ
*K. pneumoniae*	+	+	+	ـــ	ـــ	ـــ

Positive (+) = turbidity indicating growth; negative (-) = no turbidity indicating absence of growth.

**Table 3:** Growth performance of growing New Zealand (NWZ) rabbits fed diets supplemented with biological or chemical selenium nanoparticles during heat stress.

Item	Treatments (12 Rabbits/Treatments)					Pooled SEM	*p* Value
	CO	BIO25	BIO50	CH25	CH50		
Live body weight (LBW; g) at (weeks of age)							
5	661	689	663	676	666	11.65	0.513
6	773 ^b^	870 ^a^	843 ^a^	824 ^ab^	817 ^ab^	13.52	0.234
7	964	1079	1049	995	987	18.27	0.253
8	1169	1293	1230	1168	1170	19.82	0.177
9	1357	1457	1423	1334	1333	20.53	0.198
10	1524	1603	1612	1506	1484	21.72	0.221
11	1692 ^b^	1756 ^a^	1795 ^a^	1700a ^b^	1609 ^b^	25.50	0.185
12	1840 ^b^	1904 ^a^	1965 ^a^	1833 ^b^	1764 ^b^	28.45	0.219
13	1959 ^ab^	2077 ^a^	2126 ^a^	1954 ^ab^	1861 ^b^	30.34	0.028
Daily body weight gain (DBWG; g/day) at (weeks of age)							
6	20.28	24.58	25.7	21.14	21.58	0.82	0.150
7	27.28	29.86	29.44	24.4	24.28	1.21	0.440
8	29.28	30.58	25.86	24.7	26.12	0.91	0.200
9	26.86	23.4	27.58	23.72	23.26	0.79	0.240
10	23.86 ^b^	20.86 ^b^	27 ^a^	24.58 ^b^	21.56 ^b^	0.82	0.110
11	24.02 ^b^	21.86 ^b^	26.14 ^a^	27.72 ^a^	17.86 ^b^	1.19	0.060
12	21.14	21.12	24.28	19	22.16	0.76	0.290
13	17 ^bc^	23^a^	24.72 ^a^	17.26 ^bc^	13 ^c^	1.21	0.010
Feed intake (FI; g) at (weeks of age)							
6	70	80	79	73	70	1.9	0.280
7	109	93	94	83	84	3.96	0.236
8	102	114	98	103	110	3.41	0.615
9	93	88	102	87	93	2.49	0.351
10	103 ^a^	82 ^b^	107^a^	104 ^a^	93 ^a^	3.34	0.094
11	82	84	107	95	83	6.02	0.670
12	87	91	106	87	103	3.19	0.162
13	80 ^ab^	99 ^a^	93 ^ab^	74 ^ab^	66 ^c^	4.57	0.124
Feed conversion ratio (FCR; g feed/g gain) at (weeks of age)							
6	3.48	3.27	3.11	3.47	3.26	0.059	0.217
7	4.08 ^a^	3.11 ^b^	3.20 ^b^	3.40 ^b^	3.48 ^b^	0.087	0.001
8	3.48 ^c^	3.72 ^b^	3.82 ^b^	4.18 ^a^	4.22 ^a^	0.065	0.001
9	3.56	3.76	3.72	3.66	4.04	0.076	0.363
10	4.35 ^a^	3.95 ^b^	3.96 ^b^	4.23 ^ab^	4.32 ^a^	0.059	0.058
11	3.44	3.85	4.09	3.58	4.69	0.185	0.234
12	4.20	4.31	4.38	4.59	4.66	0.086	0.419
13	4.69 ^a^	4.01 ^b^	4.04 ^b^	4.43 ^ab^	4.77 ^a^	0.087	0.002

^a,b,c^ Means within a row without a common superscript letter differ at *p <* 0.05. SEM = standard error of mean. Corrections have been used for multiple comparisons. CO: control; BIO25: 25mg/kg diet of biological nano-selenium; BIO50: 50 mg/kg diet of biological nano-selenium; CH25: 25mg/kg diet of chemical nano-selenium, and CH50: 25 mg/kg diet of chemical nano-selenium.

**Table 4:** Carcass traits of growing New Zealand (NZW) rabbits fed diets supplemented with biological or chemical selenium nanoparticles during heat stress.

Item *	Treatments (12 Rabbits/Treatments)					Pooled SEM	*p* Value
%	CO	BIO25	BIO50	CH25	CH50		
Carcass	59.14 ^c^	64.38 ^a^	61.80 ^b^	60.98 ^b^	60.71 ^b^	0.49	0.001
Liver	2.80 ^a^	2.37b ^c^	2.70 ^a^	2.45 ^b^	2.30 ^c^	0.05	0.001
Lungs	0.73 ^a^	0.57 ^c^	0.60 ^bc^	0.68 ^ab^	0.66 ^abc^	0.018	0.016
Heart	0.24 ^a^	0.23 ^ab^	0.24 ^a^	0.23 ^b^	0.24 ^ab^	0.002	0.044
Kidney	0.73	0.72	0.70	0.73	0.72	0.013	0.927
Spleen	0.052	0.051	0.053	0.055	0.055	0.001	0.662

* as % of pre-slaughter weight. CO: control; BIO25: 25 mg/kg diet of biological nano-selenium; BIO50: 50 mg/kg diet of biological nano-selenium; CH25: 25 mg/kg diet of chemical nano-selenium, and CH50: 25 mg/kg diet of chemical nano-selenium. ^a, b, c^ Means within a row without a common superscript letter differ at *p <* 0.05.

**Table 5:** Blood serum metabolites inflammatory and antioxidant indicators of NZW rabbits fed diets supplemented with biological or chemical selenium nanoparticles during heat stress.

Items *	Treatments (12 Rabbits/Treatments)					Pooled SEM	*p* Value
	CO	BIO25	BIO50	CH25	CH50		
Creatinine (mg/dL)	1.46	4.15	1.12	1.26	1.35	0.60	0.500
Urea (mg/dL)	52.81 ^a^	51.11 ^ab^	45.85 ^c^	49.42 ^b^	51.34 ^ab^	0.74	0.010
GGT (U/L)	5.36 ^ab^	5.02 ^bc^	4.74 ^c^	4.95 ^c^	5.48 ^a^	0.08	0.001
TG (mg/dL)	144.78 ^a^	75.44 ^c^	73.09 ^c^	73.74 ^c^	107.33 ^b^	7.51	0.050
Total bilirubin (mg/dL)	0.88	0.89	0.82	0.83	0.89	6.01	0.310
Direct bilirubin (mg/dL)	0.21	0.19	0.20	0.20	0.19	0.00	0.270
Indirect bilirubin (mg/dL)	0.76	0.76	0.74	0.74	0.71	0.01	0.001
IL-4 (pg/mL)	0.31 ^a^	0.27 ^b^	0.28 ^b^	0.26 ^b^	0.31 ^a^	0.01	0.010
IFNγ (pg/mL)	0.18 ^b^	0.16 ^b^	0.17 ^b^	0.17 ^b^	0.20 ^a^	0.00	0.001
LZM (ng/mL)	0.22 ^b^	0.24 ^b^	0.27 ^a^	0.29 ^a^	0.22 ^b^	0.01	0.001
NO (µmol/L)	0.26 ^a^	0.20 ^c^	0.20 ^d^	0.22 ^c^	0.24 ^b^	0.01	0.001
MDA (nmol/mL)	0.31 ^a^	0.26 ^c^	0.24 ^d^	0.246 ^cd^	0.29 ^b^	0.01	0.001
SOD (U/mL)	0.29 ^a^	0.24 ^cd^	0.22 ^d^	0.25 ^c^	0.27 ^b^	0.01	0.001
GSH (ng/mL)	0.13 ^c^	0.16 ^b^	0.17 ^b^	0.19 ^a^	0.12 ^d^	0.01	0.001
CAT (ng/mL)	0.21 ^c^	0.25 ^b^	0.25 ^b^	0.29 ^a^	0.18 ^d^	0.01	0.050

* CO: control; BIO25: 25 mg/kg diet of biological nano-selenium; BIO50: 50 mg/kg diet of biological nano-selenium; CH25: 25 mg/kg diet of chemical nano-selenium, and CH50: 25 mg/kg diet of chemical nano-selenium. GGT: glutamyl transferase; TG: triglyceride; IL-4: interleukin 4; IFNγ: interferon-gamma; NO: nitric oxide; MDA: malondialdehyde; GSH: reduced glutathione; SOD: superoxide dismutase; CAT: catalase, and LZM: lysozyme. ^a, b, c, d^ Means within a row without a common superscript letter differ at *p <* 0.05.

## Supplementary Materials

The following are available online at https://www.mdpi.com/2076-2615/10/3/430/s1. Table S1: Ingredients and composition of basal diet of growing rabbits (as fed).
