# Supplementary material for: Effects of Dietary Biological or Chemical-Synthesized Nano-Selenium Supplementation on Growing Rabbits Exposed to Thermal Stress
Source: Animals (Basel). 2020 Mar 4;10(3):430. doi: 10.3390/ani10030430 (PMC7142898; doi:10.3390/ani10030430)
Supplement: Supplementary file 1 [file animals-10-00430-s001.pdf]

Supplementary material

Table S1: Ingredients and composition of basal diet of growing rabbits (as fed).

Items	Basal diet
Ingredient	%
Maize	20
Soybean meal	20
Wheat bran	16
Berseem hay	30
Barley grain	10
Molasses	2
Limestone	1
NaCl	0.5
Premix*	0.5
Calculated composition,%**	
ME, MJ/kg	7.95
Crude protein	17.50
Calcium	0.88
Available phosphorus	0.20

*Each 1 kg of premix (minerals and vitamins mixture) contains vit. A, 20,000 IU; vit. D3, 15,000 IU; vit. E, 8.33 g; vit. K, 0.33 g; vit. B1, 0.33 g; vit. B2, 1.0 g; vit. B6, 0.33 g; vit. B5, 8.33 g; vit. B12, 1.7 mg; pantothenic acid, 3.33 g;
